# Supplementary material for: Proximity superconductivity in atom-by-atom crafted quantum dots
Source: Nature. 2023 Aug 16;621(7977):60–5. doi: 10.1038/s41586-023-06312-0 (PMC10482682; doi:10.1038/s41586-023-06312-0)
Supplement: Supplementary file 1 — Supplementary Notes 1–4, including Supplementary Figs. 1–7 and Supplementary References. [file 41586_2023_6312_MOESM1_ESM.pdf]

nature portfolio

https://doi.org/10.1038/s41586-023-06312-0

Supplementary information

Proximity superconductivity in atom-by-atom crafted quantum dots

In the format provided by the authors and unedited

Supplementary information

Proximity superconductivity in atom-by-atom crafted quantum dots

Lucas Schneider, Khai That Ton, Ioannis Ioannidis, Jannis Neuhaus-Steinmetz, Thore Posske, Roland Wiesendanger, and Jens Wiebe

Correspondence to: lucas.schneider@physnet.uni-hamburg.de

Supplementary Note 1 | Characterization of the superconducting gaps of the substrate's bulk and tip

The energy resolution of scanning tunneling spectroscopy experiments is typically limited by thermal broadening at $T = 4.5$ K. This problem can be circumvented by the use of superconducting tips[1,2]. However, dI/dV spectra obtained with these tips do not directly represent the LDOS of the sample but a convolution of both the sample LDOS and the highly nonlinear tip DOS. For very low temperatures ($T \leq 1.5$ K), this can be compensated by numerical deconvolution of the data. In this work, we refrain from doing this since deconvolution is less accurate at elevated temperatures where additional, thermally activated features appear in the raw data (see, e.g., the near-zero bias peaks in Extended Data Fig. 5b or Supplementary Fig. 1). For the interpretation of the raw data, one has to keep in mind that all features are shifted to higher energies by $eV = \pm\Delta_t$. In order to reliably assign the experimental energy values to in-gap states, an accurate measurement of the tip gap energy Δ_t is thus crucial. For this, we measure dI/dV spectra at various tip-sample distances δz in a region with strong lateral confinement, and thus without in-gap MSSs, reflecting the energy gap of the proximitized Ag(111) substrate (Supplementary Fig. 1a). Note, that positive δz means approaching the tip towards the sample surface. At $eV = \pm(\Delta_s + \Delta_t)$, we find a large conductance peak due to the convolution of both coherence peaks of tip and the sample's bulk states. Moreover, another peak is found around $eV \approx 0 \approx |\Delta_s - \Delta_t|$, corresponding to thermally activated tunneling from the partially occupied coherence peak of one of the electrodes to the partially unoccupied one of the other electrode. For smaller tip-sample distances (larger δz), the intensity of the peak around zero bias increases, which can be attributed to Josephson tunneling between the two superconductors. Further, for smaller tip-sample distances, peaks at $\pm\Delta_s$ and $\pm\Delta_t$ appear (orange arrows). These can be related to Multiple Andreev Reflection (MAR) processes from one of the electrodes into the superconducting condensate of the other[3,4]. Taking all these peak energies together, we can estimate that the gaps of tip and the sample's bulk states are of the same magnitude $\Delta_s = \Delta_t = 1.35$ meV.

Supplementary Fig. 1b shows the same measurement performed inside a QD with a pair of MSSs at energies $\varepsilon_\pm$. There are the same prominent peaks as in Supplementary Fig. 1a at $eV = \pm(\Delta_s + \Delta_t)$, as well as at $\pm\Delta_s$ and $\pm\Delta_t$, for smaller tip-sample distances. Moreover, tunneling into the MSSs yields additional peaks at $\pm(\Delta_t + \varepsilon_\pm)$. While tunneling at sub-gap energies is typically assumed to be dominated by Andreev processes, the asymmetric spectral weight of the particle- and hole-like peaks (see Fig. 4 of the main text and Supplementary Fig. 4d) indicates that single electron tunneling plays a dominant role at the large junction resistances used throughout this work. Presumably, just as for Yu-Shiba-Rusinov (YSR) states, tunneling into these resonances occurs via both single electron tunneling and resonant Andreev processes[5–7]. Additionally, we find resonances at energies $\pm(\Delta_t - \varepsilon_\pm)$, indicative of thermally excited quasiparticles tunneling into the MSSs. At very small tip-sample distances, a peak at zero bias starts to rise due to Josephson tunneling. Moreover, additional peaks appear at $\varepsilon_\pm$. For YSR states, these resonances have been attributed to either an Andreev process through the YSR state or to an imperfect tip gap[4,8]. Since we use superconducting bulk Nb tips and the peak's intensity increases with δz, it is likely that Andreev processes through the MSS are responsible for the $\pm\varepsilon$ peaks. In contrast to previous works[4,8], we do not observe resonances at $\pm(\Delta_t - \varepsilon_\pm)/2$. These could either be too small in intensity to be detected or hidden within the other peaks at our experimental temperature of 4.5 K. Therefore, care should be taken in the

interpretation of weakly pronounced in-gap features. Importantly, since MAR processes and Josephson tunneling only set in at strongly reduced tip-sample distances $\delta z \gg 0$, ($\delta z = 0$ corresponds to the stabilization parameters used throughout this work), we can assume that single particle tunneling dominates the spectra shown in the main text.

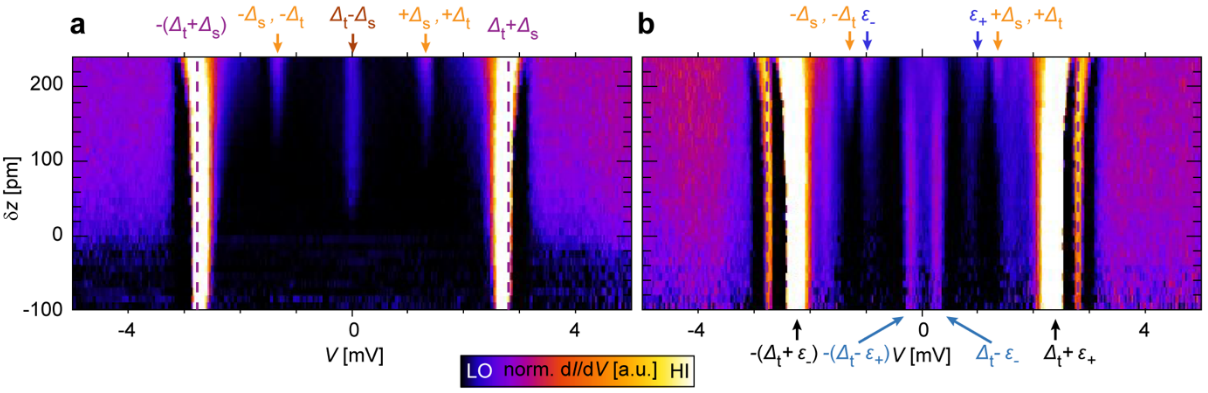

Supplementary Fig. 1 | Multiple Andreev reflection and Josephson tunneling in SIS spectra. a, d*I*/d*V* spectra measured in a region with many step edges, thus with strong lateral confinement and no MSSs for different tip-sample distances δz. Note, that positive δz means approaching the tip towards the sample surface. For normalization, the d*I*/d*V* values have been divided by the normal state conductance (i.e. at +5mV) for each δz. In the case of large tip-sample distance, we find clean SIS tunneling, whereas additional peaks appear when the tip is approached towards the sample. **b**, d*I*/d*V* spectra measured in a QD with MSSs at energies $\varepsilon_{\pm}$ for different tip-sample distances δz. Dominant peak positions are marked by colored arrows. Parameters: V_{stab} = -5 mV, I_{stab} = 1 nA, V_{mod} = 50 µV, the fixed tip height was changed by δz after stabilization.

Supplementary Note 2 | Characterization and modeling of QDs

We have measured constant-height d*I*/d*V* line profiles through all QDs with lengths ranging from L_x = 3.0 nm to L_x = 24.0 nm. The spatially resolved information from this extensive data set can be used to extract further properties of the QDs' eigenmodes, i.e. their resonance energies E_r and linewidths Γ. In Extended Data Fig. 1 of the main text, we show the full data set measured on 34 different QDs, which was used to create Fig. 2c of the main text. It can be clearly seen that the eigenmodes outside of the superconducting gap shift across E_F as the QD length L_x is tuned (the width L_y = 9.1 nm remained constant throughout this measurement series). Moreover, comparing, e.g., the QDs with L_x = 23.1 nm, L_x = 14.9 nm, and L_x = 7.3 nm, it can be seen from the corresponding figures that the eigenmodes with lower n_x at similar energies of about $V \approx$ -10 meV have larger linewidths Γ than the ones with larger n_x. We analyze the data by taking the column-wise discrete Fourier transform (FFT) of data sets like the ones presented in Extended Data Fig. 1. The absolute value of the discrete Fourier transform's n-th component $|\mathrm{FFT}_n(\mathrm{d}I/\mathrm{d}V(x))|(E)$ peaks at the eigenenergies of modes with n maxima in the data (where we define the 0th FFT component as the mean value of the data). This analysis has the benefit of filtering the signal originating from only one specific eigenmode. Supplementary Fig. 2 shows the energy-dependent 1st, 2nd, 3rd and 4th FFT components of the d*I*/d*V* line profiles in Extended Data Fig. 1. It can be seen how the n_x = 1 state (Supplementary Fig. 2a), the n_x = 2 state (Supplementary Fig. 2b), the n_x = 3 state (Supplementary Fig. 2c) and the n_x = 4 state (Supplementary Fig. 2d) shift with the QD length.

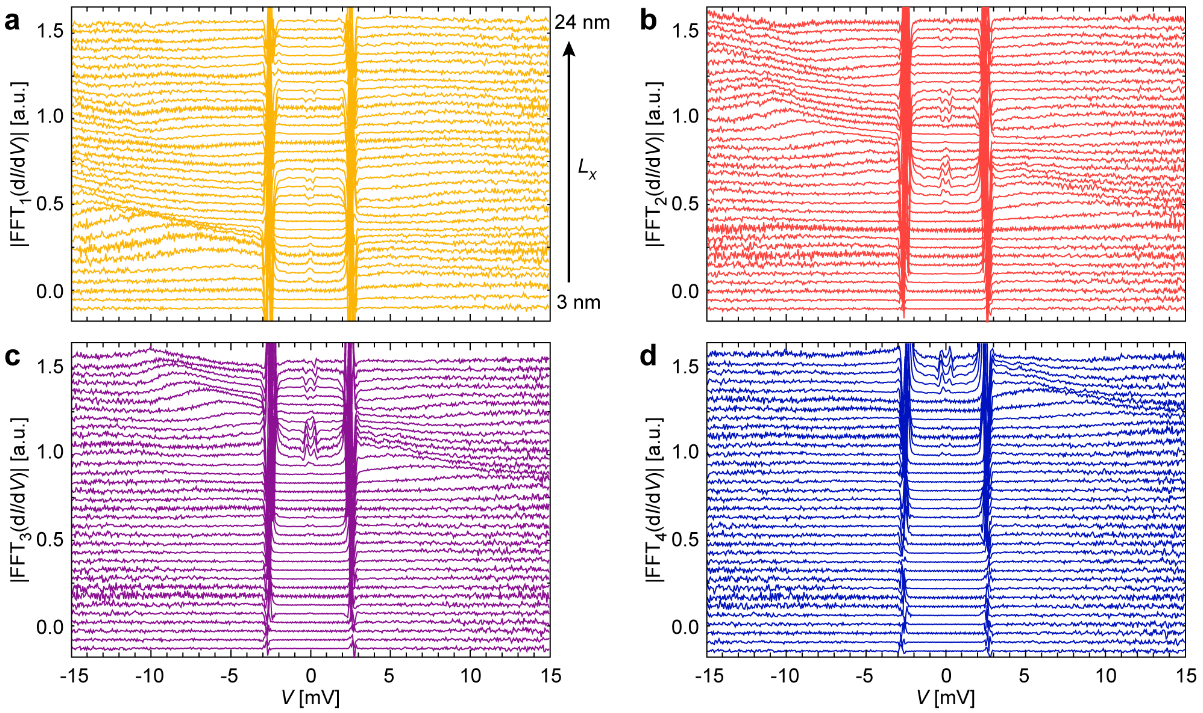

Supplementary Fig. 2 | Evolution of the nth Fourier components with QD length. a, Energy-dependent 1st Fourier component $|\mathrm{FFT}_1(\mathrm{d}I/\mathrm{d}V(x))|$ of the dI/dV line profiles in Extended Data Fig. 1. The spectra are vertically offset for clarity and are sorted by the length L_x of the QDs as indicated by the arrow on the right side of the panel. **b**, Same as panel a for the 2nd Fourier component, **c**, the 3rd Fourier component and **d**, the 4th Fourier component.

A more detailed example of the analysis is shown in Supplementary Figs. 3a-c for three selected dI/dV line profiles taken from Extended Data Fig. 1. These three examples exhibit signatures of eigenmodes around a bias voltage of $V \approx -9$ mV with $n_x = 1$, $n_x = 2$ and $n_x = 3$, respectively. Supplementary Fig. 3d shows the energy-dependent 1st, 2nd and 3rd FFT component of the respective dI/dV line profiles in Supplementary Figs. 3a-c. It can clearly be seen that the 1st component peaks at the energy where the $n_x = 1$ mode of the QD in Supplementary Fig. 3a is found. Similarly, the 2nd component peaks at the energy of the $n_x = 2$ mode in Supplementary Fig. 3b and the 3rd component does the same for the $n_x = 3$ mode in Supplementary Fig. 3c. These data points can be fitted by Lorentzian profiles

$$|\mathrm{FFT}_n(\mathrm{d}I/\mathrm{d}V(x))|(E) = A_0 + \frac{A_1}{1 + \frac{(E - E_\mathrm{r})^2}{\Gamma^2}} \tag{S1}$$

with intensity A_1, resonance energy E_r, level broadening Γ and an energy-independent contribution to the LDOS A_0. We focus on the values of E_r and Γ extracted from these fits in the following.

As we track the energy evolution of E_r in dependence on the QD length, we find a clear L_x^{-2} trend, as expected from the particle-in-a-box model (Supplementary Fig. 3e). Using Eq. (S1), the energetic positions of the eigenmodes can be fitted, enabling us to extract the surface state onset $E_0 = (-26.4 \pm 0.6)$ meV, the effective quasiparticle mass in the surface state band

m_{eff} = (0.58 ± 0.02) m_{e} and the effective hard wall offset δ = (-0.28 ± 0.15) nm. Additionally, we include the fact that all eigenenergies appear shifted to higher bias voltages by Δ_{tip} = 1.35 meV. The fitted values for E_0, m_{eff} and δ are used as an input for the particle-in-a-box model described above. The slightly reduced value for E_0 compared to previous measurements on Ag(111) bulk crystals[9] can be attributed to strain in the islands[10,11]. Small deviations from the L_x^{-2} trend can be explained by the gap opening at low energies, by deviations from the hard-wall boundary conditions of the model or by inaccuracies in the QD design since the Ag atoms can only be moved into discrete positions on a hexagonal lattice geometry. This makes it impossible to construct a perfect rectangle with arbitrary side lengths.

As a second parameter, the linewidths Γ of all eigenmodes sufficiently separated in energy from the superconducting gap can be extracted (see Supplementary Fig. 3f). The eigenmodes very close to E_{F} cannot be analyzed as their Lorentzian line shape is obscured by the presence of the superconducting gap. There is a trend towards larger energetic broadening for smaller QDs and for the eigenmodes with lower quantum numbers. The latter is already visible in the peak widths in Supplementary Fig. 3d and can be seen in Supplementary Fig. 3f in the weighted average of the Γ values for the three different eigenmodes: on average, the n_x = 3 state is the sharpest in energy, followed by n_x = 2 and, eventually, n_x = 1. These weighted average parameters and their standard deviation are used as the typical broadening values of the individual eigenmodes in Fig. 2d of the main text. The trend for broader linewidths in smaller QD structures is well-known and explained by enhanced surface-bulk scattering in small structures because of the reduced mean-free path of the surface state electrons[12,13]. However, in addition to this, it can also be seen that the eigenmodes at higher energies appear to have broader linewidths (c.f. Supplementary Fig. 6e for data measured on another QD). This may be attributed to a reduced lifetime of the surface state electrons due to enhanced electron-phonon scattering setting in at the Debye energy of the system around ±14meV[9]. Therefore, taking the weighted mean value of the linewidths at various energies is only a first approximation to the linewidth of the eigenmode if it is located at E_{F}.

It should be noted that a more sophisticated scattering theory beyond the hard-wall approximation[14] could model the eigenmodes more accurately. However, any nonlinear fit to the energy dependence of Γ would require an elaborate theory for the expected $\Gamma(E)$, which is beyond the scope of this work and is not expected to yield drastically different results. Moreover, other factors including lifetime broadening from sources other than surface-bulk scattering (e.g. electron-phonon scattering[9]) or inaccuracies in the QD design can influence the eigenmodes' widths, adding another systematic error which is almost impossible to quantitatively determine. Nevertheless, we can conclude that Γ decreases monotonously with n_x. Therefore, for our purpose of describing the shapes of individual eigenmodes, the simple model matches the data sufficiently well.

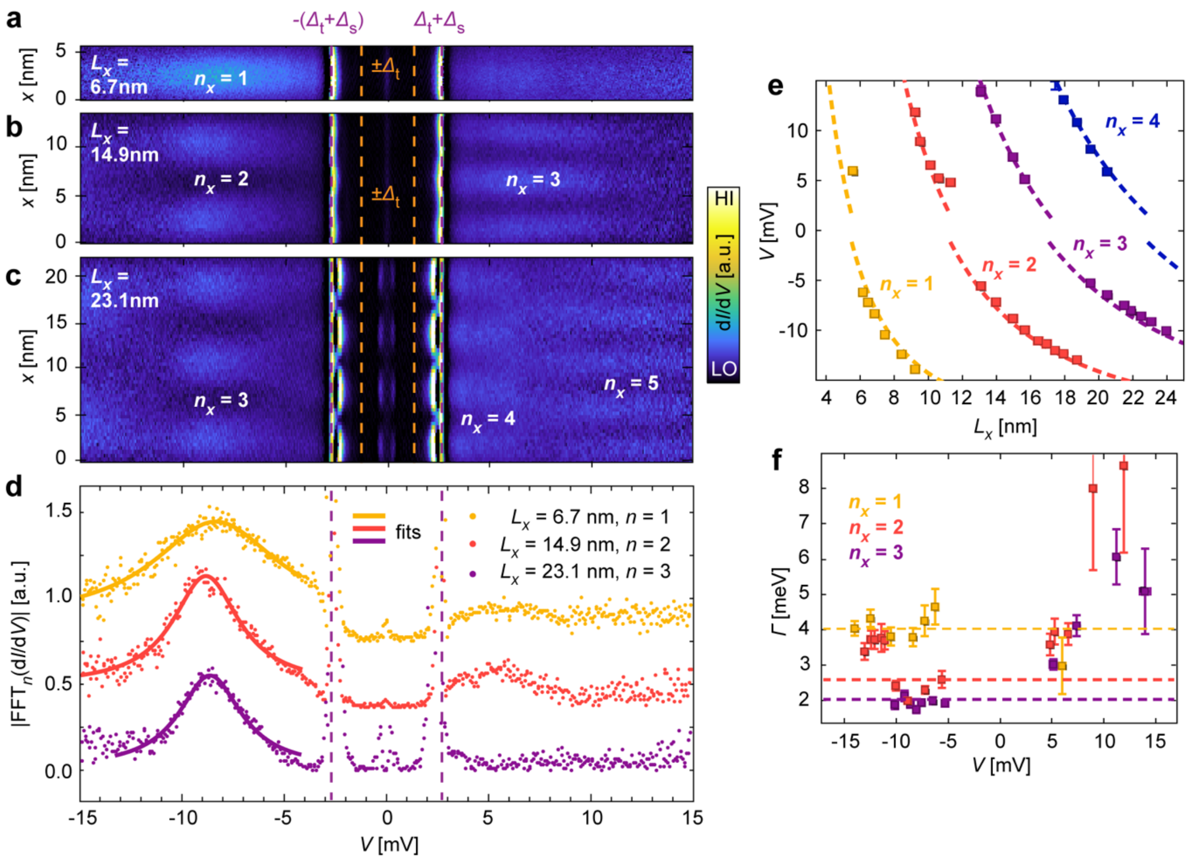

Supplementary Fig. 3 | Analysis of the eigenmodes with QD size. dI/dV line profiles measured along the central vertical axis of QDs with width L_y = 9.1 nm and length L_x = 6.7 nm (**a**), L_x = 14.9 nm (**b**) and L_x = 23.1 nm (**c**), respectively. The lines have been measured at constant tip height. The values of the tip's superconducting gap $eV = \pm\Delta_t$ and the sum $eV = \pm(\Delta_t + \Delta_s)$ of the tip gap and the proximity induced Ag bulk gap Δ_s are marked by dashed orange and purple lines, respectively. **d**, Absolute values of the 1st (yellow dots), 2nd (red dots) and 3rd (purple dots) FFT component of the respective dI/dV line profiles shown in panel a-c. The eigenmodes with n_x = 1, n_y = 1, n_x = 2, n_y = 1 and n_x = 3, n_y = 1 can be seen as peaks at the expected energies (c.f. panels a-c). Their profiles are fitted with a Lorentzian curve (solid colored lines) from which the mean energy E_r and energy broadening Γ of each individual resonance mode can be extracted. **e**, Resonance energies of the eigenmodes with n_y = 1 and n_x = {1, 2, 3, 4} for different QD lengths extracted by the fits shown exemplarily in panel d. The data points can be fitted by the expected L_x^{-2} behavior of a particle in a hard wall box (dashed lines). The discontinuity around zero energy stems from the fact that we measure with a superconducting tip and all measured energies appear at bias voltages shifted by Δ_{tip}. **f**, Resonance linewidths Γ for different QD eigenmodes. The dashed lines represent the weighted average of Γ for all eigenmodes with the same quantum numbers n_x, n_y. A trend towards sharper linewidths in larger QDs, i.e. for larger quantum numbers, can be seen.

In the second part of the analysis, we focus on the in-gap resonances found in the QD data. As shown in Fig. 2c of the main text, the in-gap states move in energy for different QD lengths. Furthermore, their particle-hole spectral weight distribution changes with QD size. To gain more insight into this effect, we project out the MSS contribution from the respective spatially resolved data sets (one example is shown in Supplementary Fig. 4a). This is done by taking the negative real part of the respective discrete FFT's n-th component as described in the previous paragraph. The negative real part of the FFT corresponds to the phase of the eigenmode's absolute square which becomes zero at both ends of the line-profiles. Thus, it corresponds to the expected confined state wavefunctions we are interested in. Very similar results are

obtained when taking the absolute value of the FFT instead. As it can be seen in Supplementary Fig. 4a, the in-gap states feature a clear spatial modulation in their wavefunction and have three maxima within the dI/dV line-profile. Accordingly, the discrete FFT's 3rd component features distinct peaks at the in-gap states' energies (Supplementary Fig. 4b). These peaks are fitted with Gaussian profiles around their maxima given by

$$-\mathrm{Re}[\mathrm{FFT}_n(dI/dV(x))](E) = A_\varepsilon \cdot e^{\frac{-(E-(\Delta_\mathrm{s}+\varepsilon))^2}{\gamma^2}} \quad \text{(S2)}$$

with intensity A_ε, resonance energy ε and broadening γ. The value Δ_s is set to the experimentally measured value of Δ_s = 1.35 meV (see Methods). As an example, Supplementary Fig. 4b shows the corresponding fits for the data presented in Supplementary Fig. 4a. From the fits, the energy evolution of ε with the QD length L_x can be traced. The resulting values are shown in Supplementary Fig. 4c for different eigenmodes with n_y = 1 and n_x = {1, 2, 3, 4}. Here, the average absolute value $\bar{\varepsilon}$ of the respective positive and negative peak energy is plotted. All values are normalized to the gap energy in the Ag bulk states Δ_s = 1.35 meV. The energies $\bar{\varepsilon}$ can clearly be seen to depend on L_x, in agreement with Fig. 2c of the main text. Notably, their minimum value ε_min is smaller for the eigenmodes with larger n_x. For a more quantitative analysis of this effect, we fit the values of $\bar{\varepsilon}(L_x)$ with yet another Lorentzian expression:

$$\bar{\varepsilon}(L_x) = \Delta_\mathrm{s}\left(1 - \frac{1 - \varepsilon_\mathrm{min}}{1 + \frac{\left(L_x - L_{\varepsilon,\mathrm{min}}\right)^2}{s^2}}\right) \quad \text{(S3)}$$

with the minimal MSS energy ε_min found at QD length $L_x = L_{\varepsilon,\mathrm{min}}$ and width s. The results are shown as dashed lines in Supplementary Fig. 4c. We use the values ε_min from this analysis of each eigenmode for the comparison with the level broadening Γ in Fig. 2d of the main text.

In addition to this effect, the in-gap states are found to have a varying ratio of particle- and hole-component in their spectral weight measured in tunneling spectroscopy, i.e. the unoccupied in-gap state appears to be higher in intensity than the occupied in-gap state for $E_\mathrm{r} > 0$ and vice versa for $E_\mathrm{r} < 0$. This peak asymmetry in spectral weight of the in-gap states can be analyzed in terms of the Bogoliubov mixing angle (Eq. (3) of the main text). The results are shown in Supplementary Fig. 4d. In the experimental data, we find a value of $\theta_\mathrm{B} \approx \pi/4$ whenever $\bar{\varepsilon} \approx \varepsilon_\mathrm{min}$ ($E_\mathrm{r} \approx 0$), as indicated by the arrows in Supplementary Fig. 4d. This finding further supports the interpretation that ε_min equals the induced superconducting pairing Δ_ind in the QD resonance level. For QDs with decreasing L_x (with resonance levels $E_\mathrm{r} > 0$), θ_B increases, indicating a dominant hole-character of the Bogoliubov quasiparticles. Conversely, θ_B decreases for QDs with increasing L_x ($E_\mathrm{r} < 0$), i.e. the quasiparticles feature a stronger particle-component. This trend is found consistently for all eigenmodes (see Supplementary Fig. 6d for another example) and qualitatively agrees well with the theoretical model for resonance scattering (see Fig. 3 and Methods of the main text).

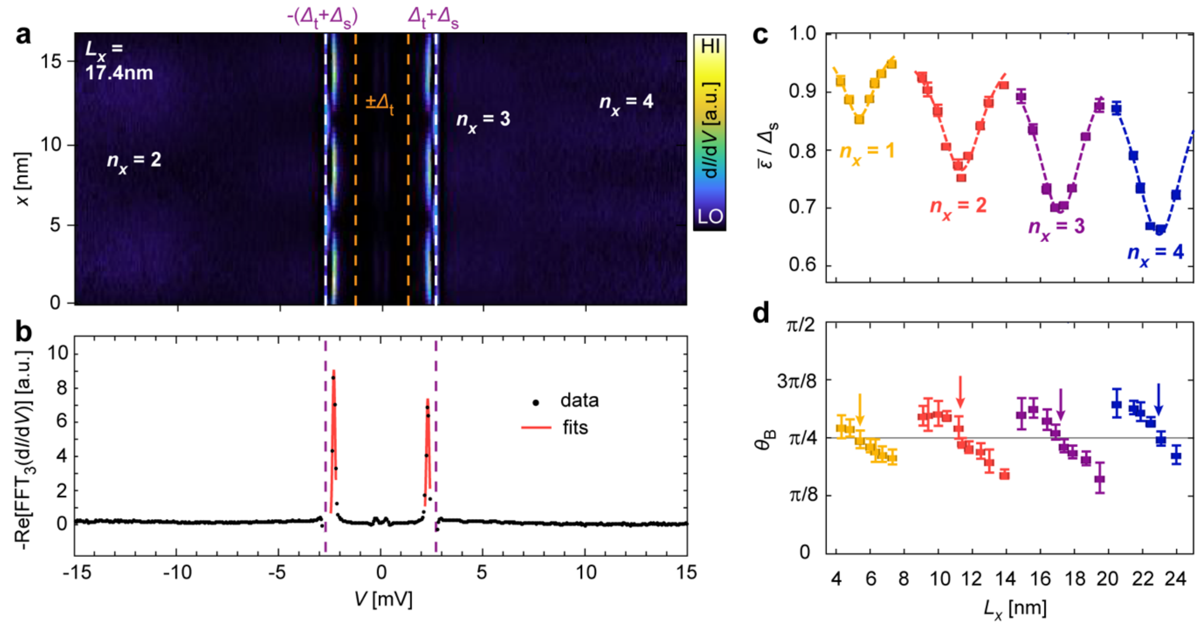

Supplementary Fig. 4 | Fitting of the in-gap states. a, d*I*/d*V* line-profile measured along the central vertical axis of a QD with length L_x = 17.4 nm and width L_y = 9.1 nm. The line has been measured at constant tip height. The values of the tip's superconducting gap $eV = \pm\Delta_t$ and the sum $eV = \pm(\Delta_t + \Delta_s)$ of the tip gap and the proximity induced Ag bulk gap Δ_s are marked by dashed orange and white lines, respectively. **b**, Negative real part of the 3rd FFT component of the d*I*/d*V* line-profile shown in panel a. The MSSs with n_x = 3 can be clearly seen as peaks at the expected energies (c.f. panel a). Their profile is fitted with Eq. (S2) (solid lines) from which the mean energy $\pm(\Delta_t + \varepsilon)$ and height A_ε of each individual peak can be extracted. **c**, Energies $\bar{\varepsilon}$ (average absolute value of the respective positive and negative peak energies) of the MSSs in QDs of varying length L_x extracted from a series of fits as in panel b. The colored labels denote the corresponding quantum numbers n_x of the relevant eigenmodes. Dashed lines show the respective fits of the data to Eq. (S3). **d**, Bogoliubov angle θ_B of the MSSs extracted from fits as in panel b. The colored arrows mark the positions of the MSSs' minimal energy $\bar{\varepsilon} \approx \varepsilon_{min}$.

Supplementary Note 3 | Machida-Shibata states in regions with strong confinement naturally occurring on Ag(111) islands

The emergence of MSSs is not only observed in artificially constructed QDs but also in naturally occurring areas on the islands in the presence of step edges. Some examples are shown in Supplementary Fig. 5. While most of the Ag(111) islands are defect free (see Extended Data Figs. 5 and 6), some still exhibit screw dislocations and step edges as a consequence of strained growth on a mismatched substrate[11]. This leads to natural confinement of the surface state quasiparticles. For small regions (see, e.g., the small terrace marked by a yellow line in Supplementary Fig. 5a), there are only few in-gap states (Supplementary Fig. 5f) as only a small number of surface state modes is energetically located close to E_F. More in-gap modes at various energies are found on larger terraces, e.g. the narrow terrace marked by the blue rectangle or the wedge-shaped terrace marked in green (c.f. Supplementary Figs. 5a and d,e). Examples of d*I*/d*V* maps obtained at in-gap energies are shown in Supplementary Figs. 5b and c. The in-gap states still follow the periodicity of the quasiparticle scattering states of the Ag surface state, indicating the close relation to the surface state modes.

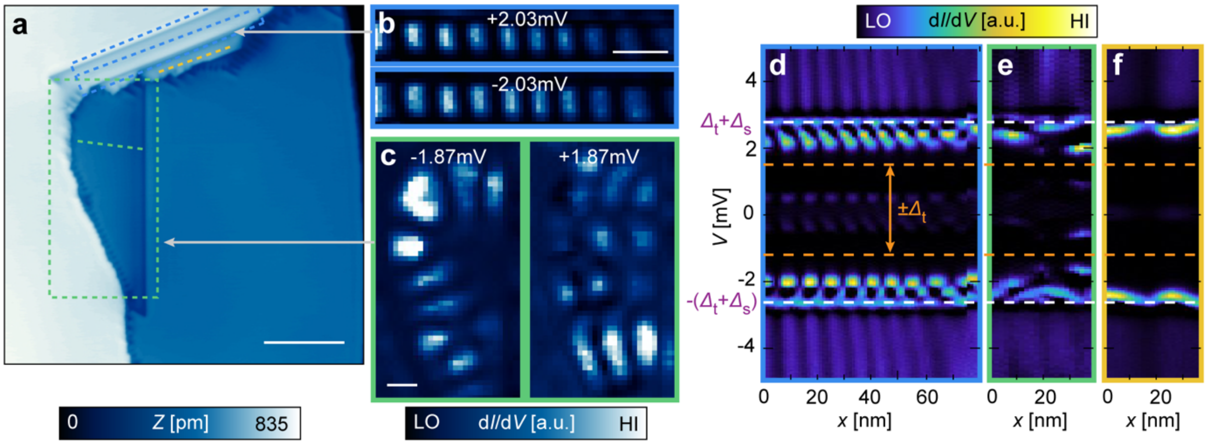

Supplementary Fig. 5 | MSSs in naturally confined regions. a, Constant-current STM image of a strained Ag(111) area featuring several step edges and screw dislocations. The white bar corresponds to 20 nm. **b**, d*I*/d*V* grid of the region marked by the blue dashed rectangle in panel a evaluated at in-gap energies of ±2.03mV. The white bar corresponds to 10 nm. **c**, d*I*/d*V* grid of the region marked by the green dashed rectangle in panel a evaluated at in-gap energies of ±1.87mV. The white bar corresponds to 10 nm. **d**, d*I*/d*V* line-profile measured on the long and narrow terrace in panel a along the blue dashed line. The values of the tip's superconducting gap $eV = \pm\Delta_t$ and the sum $eV = \pm(\Delta_t + \Delta_s)$ of the tip gap and the proximity induced Ag bulk gap Δ_s are marked by dashed orange and white lines, respectively. **e**, d*I*/d*V* line-profile measured on the wedge-shaped terrace in panel a along the green dashed line. **f**, d*I*/d*V* line-profile measured on the small terrace in panel a along the yellow dashed line. Parameters: V = 100 mV, I = 1 nA for panel a; V_{stab} = -20 mV, I_{stab} = 5 nA, V_{mod} = 100 µV for panels b, d and f; V_{stab} = 5 mV, I_{stab} = 1 nA, V_{mod} = 50 µV for panels c and e.

The narrow terraces can further be exploited to repeat the QD experiment from the main text using the linear step edges as two naturally defined walls. Additionally, Ag atoms are prepared on the terrace as described in the Methods section. These atoms can form the walls of a QD and can be laterally moved to alter the confinement conditions in the QD (see Supplementary Fig. 6a). d*I*/d*V* line-spectra along the terrace (Supplementary Fig. 6b) reveal periodically modulated in-gap states in addition to the confined states at energies outside the gap. The energetic position of the confined states as well as of the in-gap states changes with QD length (Supplementary Fig. 6c, c.f. Fig. 1f in the main text). This change follows the same systematic trend as found in Fig. 2c of the main text (Supplementary Fig. 6d): whenever a QD eigenmode crosses E_F, in-gap states at particle-hole symmetric energies are found. For larger QDs, their minimal energy moves further into the gap (c.f. Fig. 2c of the main text and Supplementary Fig. 4c). As for the fully artificial QDs described in the main text and in Supplementary Note 2, we can relate the widths of the eigenmodes outside of the gap to the minimal energy of the in-gap states. The analysis of the eigenmode linewidths Γ is shown in Supplementary Fig. 6e. It corroborates the trend of decreasing linewidth with increasing quantum number of the eigenmode, i.e. the n_x = 1 state has the broadest linewidth, followed by n_x = 2, 3, 4 and, eventually, 5. Similarly, the minimal energies $\varepsilon_{\mathrm{min}}$ of the in-gap states is evaluated using the same method as described in Supplementary Note 2 (see Supplementary Fig. 4). The resulting data points for n_x = {1, 2, 3, 4, 5} have been added to Fig. 2d of the main text and agree reasonably well with the trend predicted by the resonance scattering model. This suggests the same underlying physics as in the artificial QDs exclusively built by manipulated Ag atom walls shown in the main text.

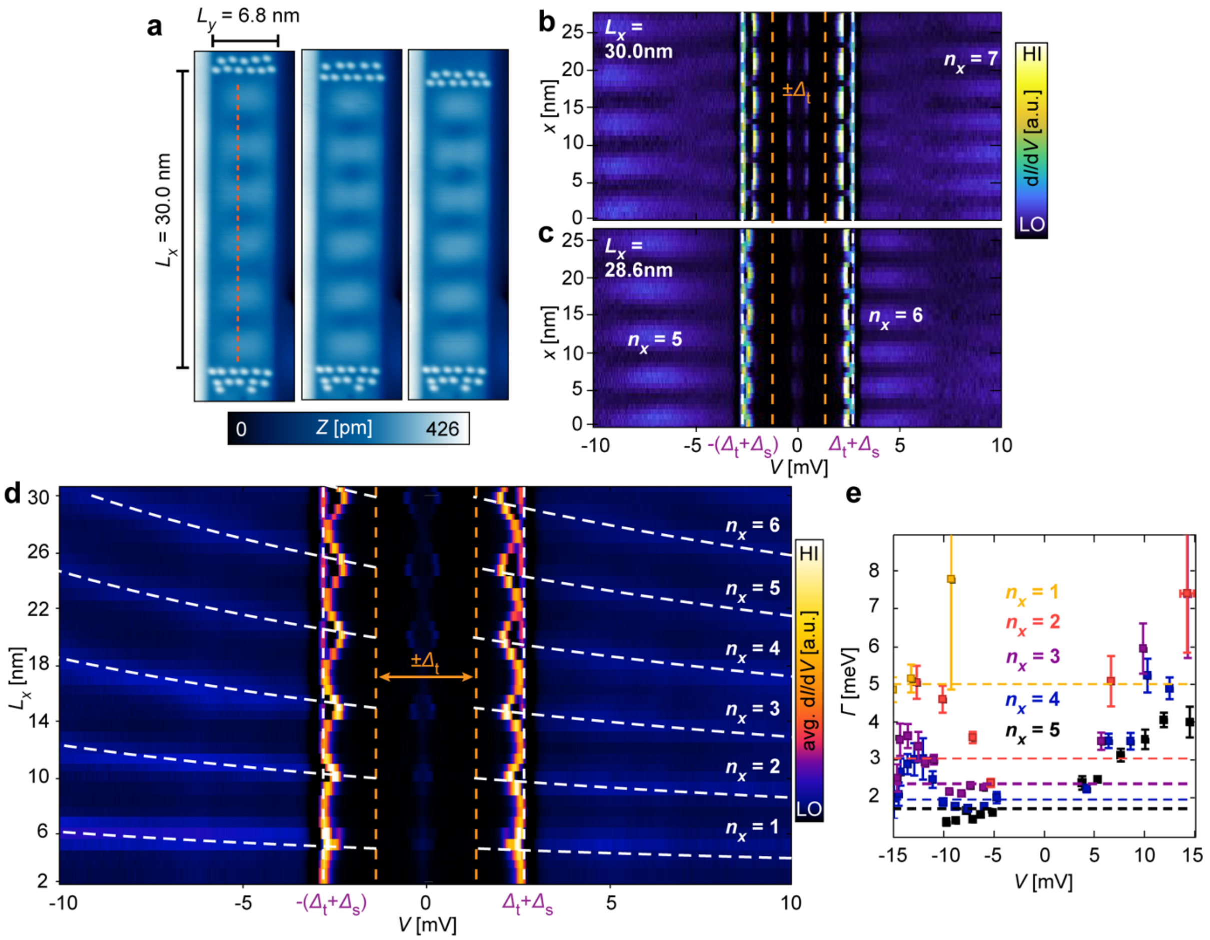

Supplementary Fig. 6 | Tuning of the MSSs on a quasi-1D terrace with additional Ag atoms. a, Constant-current STM images of the quasi-one-dimensional terrace marked by the blue rectangle in Supplementary Fig. 5a with additional Ag adatoms creating boundaries of a rectangular QD with tunable length L_x but fixed width L_y. **b**, dI/dV line-profile measured along the red dashed line marked in panel a, i.e. on a QD with length 30.0 nm. The line has been measured at constant tip height. **c**, dI/dV line-profile measured along the same vertical line as in panel b after one of the QD walls has been moved and the QD size has been changed. In both panels b and c, signatures of the eigenmodes with $n_y = 1$ and $n_x = \{5, 6, 7\}$ are marked at energies outside the gap. The values of the tip's superconducting gap $eV = \pm\Delta_t$ and the sum $eV = \pm(\Delta_t + \Delta_s)$ of the tip gap and the proximity induced Ag bulk gap Δ_s are marked by dashed orange and white lines, respectively. **d**, Evolution of averaged dI/dV spectra from dI/dV line-profiles measured along the central axis of the terrace vertically (along lines equivalent to the one marked in panel a) as a function of the QD length L_x. The white dashed lines mark the evolution of the eigenmodes with $n_y = 1$ and $n_x = \{1, 2, 3, 4, 5, 6\}$ obtained from fitting the dI/dV spectra at energies outside of the gap (analogous to the analysis in Supplementary Note 2). **e**, Resonance linewidths Γ for different eigenmodes appearing at bias voltages V evaluated by the method described in Supplementary Note 2. The colored horizontal solid lines represent the weighted average of Γ for all eigenmodes with the same quantum numbers n_x, n_y. The trend towards sharper linewidths in larger QDs can be seen for each energy interval. In addition, the levels close to E_F appear to be sharper than the ones at higher energies. Parameters: V = 5 mV, I = 1 nA for panel a; V_{stab} = 15 mV, I_{stab} = 4 nA, V_{mod} = 50 µV for panel b.

Supplementary Note 4 | Real-space tight-binding model of Ag QDs in proximity to a superconductor

In this Supplementary Note, we employ a three-dimensional (3D) tight-binding model of the actual geometry of the setup and find qualitatively the same behavior as the analytic theory presented the main text. This study corroborates the interpretation of the QD eigenmodes obtaining superconducting pairing. Furthermore, we resolve the spatial structure of the QD eigenmodes, their dependence on the QD's length and find that they are consistent to the particle-in-the-box model presented in the main text.

The tight-binding model follows the geometry sketched in Supplementary Fig. 7a, consisting of one superconducting (SC) layer (dark gray, triangular lattice), modeling the SC substrate, one normal conducting layer (gray, triangular lattice), representing the Ag(111) surface states (SS), and a QD built of non-magnetic adatom sites (dark blue) arranged on top of the surface layer. We assume a rectangular shape of the QD of dimensions L_x x L_y and the adatom arrangement is chosen such that the atoms occupy the nearest available hollow adsorption sites along the rim of the rectangle. The SC layer is weakly coupled to the surface layer by a hopping term $t_{\text{SC-SS}}$ (blue lines in Supplementary Fig. 7a).

The model is constructed by the following Hamiltonians:
The SC layer consists of a triangular lattice of 150 x 150 sites with a lattice constant *a* set to *a* = 0.25 nm and is described by

$$\mathcal{H}_{\mathrm{SC}} = \sum_{i_{\mathrm{sc}}} c_i^\dagger(-\mu_{\mathrm{SC}}\tau_z \otimes \sigma_0 + \Delta_{\mathrm{s}}\tau_x \otimes \sigma_0)c_i + \sum_{\langle i_{\mathrm{sc}}, j_{\mathrm{sc}}\rangle} \left(c_i^\dagger(-t_{\mathrm{SC}}\tau_z \otimes \sigma_0)c_j + \mathrm{h.c.}\right), \quad \text{(S4)}$$

using the basis $c_i = \left(c_{i\uparrow}, c_{i\downarrow}, c_{i\downarrow}^\dagger, -c_{i\uparrow}^\dagger\right)^{\mathrm{T}}$. Here, $c_{i,\sigma}^\dagger$ and $c_{i,\sigma}$ are the creation and annihilation operators of fermionic states with spin σ at site *i*, $\langle i, j\rangle$ indicates the summation over nearest neighbor sites, and i_{SC} indicates that the index i in the corresponding sum refers to sites on the superconductor. μ_{SC} is the chemical potential in the SC layer, Δ_{s} is the superconducting order parameter and t_{SC} is the hopping strength within the layer. Moreover, τ_i and σ_i are the Pauli matrices in particle-hole- and spin-space, respectively, and $\otimes$ represents the Kronecker product.

Similarly, the surface layer consists of 150 x 150 sites on a triangular lattice and is described by

$$\mathcal{H}_{\mathrm{SS}} = \sum_{i_{\mathrm{surface}}} c_i^\dagger(-\mu_{\mathrm{SS}}\tau_z \otimes \sigma_0)c_i + \sum_{\langle i_{\mathrm{surface}}, j_{\mathrm{surface}}\rangle} \left(c_i^\dagger(-t_{\mathrm{SS}}\tau_z \otimes \sigma_0)c_j + \mathrm{h.c.}\right), \quad \text{(S5)}$$

with the chemical potential μ_{SS} and the intra-layer hopping strength t_{SS}. Notably, no intrinsic superconducting pairing is assumed.

The on-site potential of the adatoms is given by

$$\mathcal{H}_{\text{ad}} = \sum_{i_{\text{ad}}} c_i^{\dagger}(-\mu_{\text{ad}}\tau_z \otimes \sigma_0)c_i. \tag{S6}$$

The SC and the surface layer are coupled by

$$\mathcal{H}_{\text{SC-SS}} = \sum_{\langle i_{\text{SC}}, j_{\text{surface}} \rangle} c_i^{\dagger}(-t_{\text{SC-SS}}\tau_z \otimes \sigma_0)c_j + \text{h.c.}, \tag{S7}$$

with the coupling strength $t_{\text{SC-SS}}$. Moreover, the adatoms are coupled to the surface layer by

$$\mathcal{H}_{\text{ad-SS}} = \sum_{\langle i_{\text{ad}}, j_{\text{surface}} \rangle} c_i^{\dagger}(-t_{\text{SS}}\tau_z \otimes \sigma_0)c_j + \text{h.c.}. \tag{S8}$$

We employ periodic boundary conditions and use the following parameters, which accurately reproduce the experimental data of Supplementary Note 2 and is consistent with the well-known band dispersion of the Ag(111) surface state[9] adjusted to the silver islands: $t_{\text{SC}} = t_{\text{SS}} = 1100$ meV, $\mu_{\text{ad}} = -110$ meV, $\mu_{\text{SC}} = \mu_{\text{SS}} = -6545$ meV. Additionally, we choose $\Delta_{\text{s}} = 25$ meV in the superconducting layer.

We construct the full Hamiltonian

$$\mathcal{H} = \mathcal{H}_{\text{SC}} + \mathcal{H}_{\text{SS}} + \mathcal{H}_{\text{ad}} + \mathcal{H}_{\text{SC-SS}} + \mathcal{H}_{\text{ad-SS}} \tag{S9}$$

within the *Kwant* framework[15]. The 2200 lowest energy eigenstates of $\mathcal{H}$ are calculated for the evaluation of the energy-dependent LDOS(E, x) within the layer $l_{1,2}$. The latter is done by summing over all pairs of the resulting eigenvalues E_i and eigenvectors ψ_i:

$$\text{LDOS}(E, x) \approx \sum_{i \in l_{1,2}} |\psi_i(x)|^2 e^{-(E-E_i)^2/\delta E_{th}{}^2} \tag{S10}$$

with $\delta E_{th} = 0.025\Delta_{\text{s}}$ simulating the experimental thermal broadening relative to the superconducting gap. The energy-dependent DOS inside the QDs is calculated by restricting the sum in Eq. (S10) to the SS layer and summing over sites x that are more than three sites away from the QD's boundary.

We present the energy-dependent DOS evaluated inside a QD of varying length L_x with a fixed width L_y of 30 sites (corresponding to 7.5 nm) in Supplementary Fig. 7b, where we set the coupling to the SC layer to zero in order to clearly resolve the QD's eigenmodes. The eigenmodes are following the well-known L_x^{-2} trend and shift continuously through the Fermi energy E_F as a function of the QD length. When a small coupling to the SC layer is considered (Supplementary Fig. 7c), the eigenmodes acquire particle-hole mixing and show an anticrossing behavior close to E_F. This is consistent with the findings of the resonance scattering model presented in the main text (c.f. Fig. 3) and shows how the QD's eigenmodes modes acquire superconducting pairing. Within the tight-binding framework, we are additionally able to depict the spatial distribution of the QD's eigenstates. Supplementary Fig. 7d shows two examples for the LDOS in QDs of different lengths (L_x = 25 sites and L_x = 40

sites, corresponding to 6.25 and 10.0 nm, respectively), where the energy is integrated over an energy window centered around the respective eigenenergy of the QD, as indicated above the panels.

It should be mentioned that the MSSs derived from the resonance scattering Hamiltonian in the main text are accompanied by broadened impurity resonances outside of the superconducting gap $\pm\Delta_\mathrm{S}$. This effect cannot be resolved directly by a tight-binding model because of strong finite-size effects typical for these models. However, it can be shown that the level broadening manifests itself differently: if the coupling to the SC layer is zero, the [n_x, n_y] = [1, 1] eigenmode in a QD of 25x30 sites (corresponding to 6.25 x 7.5 nm^2, Supplementary Fig. 7d, left panel) only has spectral intensity at in-gap energies (i.e., in a narrow energy window around the eigenvalue of this state, c.f. Supplementary Fig. 7b) but not at energies outside of the gap (Supplementary Fig. 7e, left panel). In contrast, the spatial distribution of this eigenmode is visible both inside and outside of the gap if the coupling is finite (Supplementary Fig. 7e, right panel). This is in agreement with the interpretation that the original eigenmode becomes broadened by finite coupling to a host (c.f. Fig. 2c of the main text as well as Supplementary Figs. 4a and 6d, showing the coexistence of sharp MSSs and the broader resonance level outside the gap).

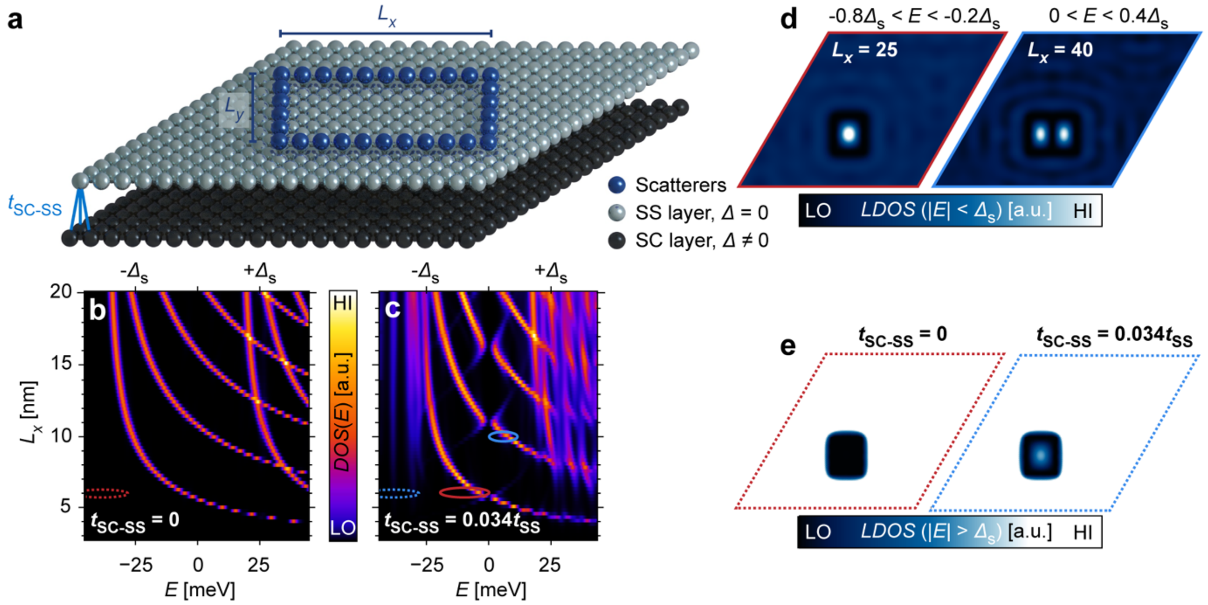

Supplementary Fig. 7 | Tight-binding modelling of proximity-induced pairing in QDs. a, Sketch of the geometry considered in the model of Supplementary Note 4. Each sphere represents one fermionic site with spin-1/2. While the SC layer features finite pairing terms, the bare SS layer is a normal metal. Pairing in the SS layer is induced via proximity coupling $t_{\mathrm{SC-SS}}$ between the layers (indicated by blue lines). The QD is built by scattering sites on the surface aligned in a rectangular geometry of length L_x and width L_y. **b**, Evolution of the energy-dependent DOS evaluated within the QD for zero coupling to the SC layer and varying length L_x and fixed width L_y of 7.5 nm (= 30 sites). **c**, Same as panel c but with a finite coupling to the SC layer, $t_{\mathrm{SC-SS}} = 0.034 t_{\mathrm{SS}}$. The QD eigenmodes aquire a proximity-induces gap. **d**, Spatially resolved LDOS patterns in the SS layer evaluated at in-gap energies integrated over the energy ranges indicated above the two panels for QDs of 25x30 (= 6.25 x 7.5 nm^2, left panel) and 40x30 sites (= 10.0 x 7.5 nm^2, right panel), respectively (again $t_{\mathrm{SC-SS}} = 0.034 t_{\mathrm{SS}}$). The eigenmodes of the rectangular QDs are prominently visible. The frame colors of the panels correspond to the solid ellipses in panel c, highlighting which of these eigenstates are shown. **e**, Spatially resolved LDOS at

energies outside the superconducting gap (integrated from -$2.00\Delta_s$ to $-1.12\Delta_s$) for zero (left panel) and finite (right panel) coupling to the SC layer. The frame colors of the panels correspond to dashed ellipses in panels b and c. For zero coupling, the states confined to the interior region of the QD are energetically sharp whereas they acquire a finite width when coupled to the bath. The LDOS signatures of the QD eigenmodes become visible at out-of-gap energies only for a finite coupling to the SC layer, see right panel.

References

1. Pan, S. H., Hudson, E. W. & Davis, J. C. Vacuum tunneling of superconducting quasiparticles from atomically sharp scanning tunneling microscope tips. *Appl. Phys. Lett.* **73**, 2992–2994 (1998).
2. Ruby, M., Heinrich, B. W., Pascual, J. I. & Franke, K. J. Experimental Demonstration of a Two-Band Superconducting State for Lead Using Scanning Tunneling Spectroscopy. *Phys. Rev. Lett.* **114**, 157001 (2015).
3. Ternes, M. *et al.* Subgap structure in asymmetric superconducting tunnel junctions. *Phys. Rev. B* **74**, 132501 (2006).
4. Randeria, M. T., Feldman, B. E., Drozdov, I. K. & Yazdani, A. Scanning Josephson spectroscopy on the atomic scale. *Phys. Rev. B* **93**, 161115 (2016).
5. Ruby, M. *et al.* Tunneling Processes into Localized Subgap States in Superconductors. *Phys. Rev. Lett.* **115**, 087001 (2015).
6. Peters, O. *et al.* Resonant Andreev reflections probed by photon-assisted tunnelling at the atomic scale. *Nat. Phys.* **16**, 1222–1226 (2020).
7. Thupakula, U. *et al.* Coherent and Incoherent Tunneling into Yu-Shiba-Rusinov States Revealed by Atomic Scale Shot-Noise Spectroscopy. *Phys. Rev. Lett.* **128**, 247001 (2022).
8. Farinacci, L. *et al.* Tuning the Coupling of an Individual Magnetic Impurity to a Superconductor: Quantum Phase Transition and Transport. *Phys. Rev. Lett.* **121**, 196803 (2018).
9. Grothe, S. *et al.* Quantifying Many-Body Effects by High-Resolution Fourier Transform Scanning Tunneling Spectroscopy. *Phys. Rev. Lett.* **111**, 246804 (2013).
10. Tomanic, T., Schackert, M., Wulfhekel, W., Sürgers, C. & Löhneysen, H. V. Two-band superconductivity of bulk and surface states in Ag thin films on Nb. *Phys. Rev. B* **94**, 220503 (2016).
11. Tomanic, T. *et al.* Local-strain mapping on Ag(111) islands on Nb(110). *Appl. Phys. Lett.* **101**, 063111 (2012).
12. Crampin, S., Jensen, H., Kröger, J., Limot, L. & Berndt, R. Resonator design for use in scanning tunneling spectroscopy studies of surface electron lifetimes. *Phys. Rev. B* **72**, 035443 (2005).
13. Heers, S., Mavropoulos, P., Lounis, S., Zeller, R. & Blügel, S. Lifetime reduction of surface states at Cu, Ag, and Au(111) caused by impurity scattering. *Phys. Rev. B* **86**, 125444 (2012).
14. Fiete, G. A. & Heller, E. J. Colloquium : Theory of quantum corrals and quantum mirages. *Rev. Mod. Phys.* **75**, 933–948 (2003).
15. Groth, C. W., Wimmer, M., Akhmerov, A. R. & Waintal, X. Kwant: a software package for quantum transport. *New J. Phys.* **16**, 063065 (2014).
